# Cortical Correlates of Impulsive Aggressive Behavior in Pediatric Bipolar Disorder

**DOI:** 10.3389/fpsyt.2021.674707

**Published:** 2021-07-21

**Authors:** Alessio Simonetti, Sherin Kurian, Johanna Saxena, Christopher D. Verrico, Antonio Restaino, Marco Di Nicola, Jair C. Soares, Gabriele Sani, Kirti Saxena

**Affiliations:** ^1^Menninger Department of Psychiatry and Behavioral Sciences, Baylor College of Medicine, Houston, TX, United States; ^2^Department of Neuroscience, Section of Psychiatry, Fondazione Policlinico Universitario “Agostino Gemelli” Istituto di Ricovero e Cura a Carattere Scientific (IRCCS), Rome, Italy; ^3^Department of Psychiatry, Texas Children's Hospital, Houston, TX, United States; ^4^Department of Neuroscience, Section of Psychiatry, Università Cattolica del Sacro Cuore, Rome, Italy; ^5^Department of Psychiatry and Behavioral Sciences, University of Texas Health Science Center, Houston, TX, United States

**Keywords:** pediatric bipolar disorder, impulsive aggression, cortical thickness, anger, hostility

## Abstract

**Background:** Impulsive aggression represents a frequent characteristic of pediatric bipolar disorder (PBD). Cortical alterations associated with impulsive aggression and its multiple facets have not been investigated yet in youth with bipolar disorder.

**Aim:** To investigate the relationship between cortical thickness and facets of impulsive aggression in youth with PBD.

**Materials and Methods:** Twenty-three youth with PBD and 23 healthy controls (HC) were administered the aggression questionnaire (AQ) and underwent 3T magnetic resonance imaging scan. Cortical thickness was assessed with FreeSurfer. Canonical correlation analyses were used to investigate the relationship between AQ total and subscale scores and cortical thickness in youth with PBD.

**Results:** Youth with PBD had increased scores in the subscales of AQ-anger and AQ-hostility and cortical thinning in in areas belonging to the affective network (AN), frontoparietal network (FPN) and cingulo-opercular network (CON), i.e., right rostral anterior cingulate, right caudal anterior cingulate, right lateral orbitofrontal, right medial orbitofrontal, left and right inferior parietal, left posterior cingulate, left and right supramarginal left lingual cortices. Greater thickness in these networks positively correlated with the AQ-hostility subscale and negatively correlated with AQ-anger subscale.

**Conclusions:** The opposite patterns observed between areas belonging to AN, FPN, CON, and the two facets of IA, namely anger and hostility, corroborate clinical findings supporting the different nature of these two constructs.

## Introduction

Aggression is defined as behaviors in which physical force is used with the intent to harm or damage (1). Aggression can be purposeful, unemotional and controlled (i.e., predatory aggression), or manifest itself in reaction to a perceived threat in an emotionally charged situation [i.e., impulsive aggression (IA)] (2). IA manifests itself verbally or physically (3) and is the endpoint of a complex psychopathological cascade starting from hostility. Hostility is a cognitive construct defined as an attitude that involves the dislike and the negative evaluation of others and others' intentions (4). Such attitudinal trait predisposes individuals to respond to external cues with frequent and intense angry feelings and related behavior (4). Therefore, hostility preludes anger, i.e., a multifaceted construct consisting of physiological (general sympathetic arousal), cognitive (irrational beliefs, automatic thoughts, inflammatory imagery), phenomenological (subjective awareness and labeling of angry feelings) and behavioral (facial expressions and verbal/ behavioral anger expression strategies) facets (5–7) Anger in turn serves as a cue for developing physical or verbal aggression (8). IA is present across psychiatric diagnoses (9), but is especially present in bipolar disorder (BD) (10).

BD is a chronic and debilitating illness characterized by cyclic alternations of phases of elated mood, increased energy, reduced need for sleep, increased self-esteem, flight of ideas, distractibility and increased involvement in goal directed activity and, phases of depressed mood, feelings of guilt, motor retardation or restlessness, insomnia, and suicidal ideation (11). The onset of BD is usually in late adolescence/early adulthood, although it can also affect children (12, 13). Juvenile-onset BD, or pediatric bipolar disorder (PBD) has a more severe illness course than adult-onset BD, with higher rates of chronicity, cyclicity, and poorer prognosis (14). IA is noted to be a frequent characteristic in individuals with PBD. Indeed, Hernandez et al. (15), reported IA to be present in more than 50% of 7-year olds with PBD. In young adolescents, these rates (16) almost reached 40%. Rates of IA are higher in PBD compared with ADHD (17) or juvenile depression (16), and have been addressed as one of the most common “red flags” that should alert clinicians for the presence of BD in children and adolescents (13). Papolos et al. (18) included IA among a cluster of symptoms identifying a specific phenotype of PBD characterized by a poor prognosis. Furthermore, our group has shown that IA is associated with impairment in specific cognitive domains, such as affective processing and executive functions (19). Finally, IA is regarded as a common and major public health issue that increases the risk of substance abuse, suicidal behaviors, incarceration and violence in adulthood (20).

Despite the pivotal importance of IA in youth with PBD, its neural correlates have been poorly understood. Specifically, alterations in the cortical sheet, which changes with age (21, 22), in neurological (21, 23) and psychiatric disorders (24, 25), and represents a promising *in-vivo* biomarker proven useful for diagnosis (26), has not been investigated yet. The extant literature on this topic has mainly focused on healthy youth (27–32) with additional reports in youth with autism spectrum disorders (33) conduct disorder (CD) (30) or attention-deficit and hyperactivity disorder (ADHD) (34). These findings highlighted that, independently from the diagnosis, IA is related to thinning of areas of frontal and parietal lobes. Specifically, cortical thinning was reported in areas belonging to the affective network, i.e., an extensive network involved in mood experience and regulation; and to the control network, i.e., a hub of interrelated networks involved in monitoring performances and control impulses and behavior (35–39). Among all the areas involved, thinning in the anterior cingulate cortex (ACC), posterior cingulate cortex (PCC), orbitofrontal cortex (OFC), supramarginal gyrus (SMG) and precuneus were repeatedly reported. Furthermore, all the aforementioned studies limited the analyses of the neural correlates of IA to the whole construct, and to date, possible differences in cortical thickness and all the facets of IA need to be investigated.

The aim of this study is to evaluate the relationship between cortical thickness, IA, and its facets in youth with PBD. Given the transdiagnostic nature of IA, and its relationship with thinning in areas belonging to the affective network and to the control network, we expected youth with PBD to show inverse correlations between the several facets of IA and the cortical thickness of areas belonging to these networks. More specifically, given the most robust findings regarding thinning of ACC, PCC, OFC, SMG, and the precuneus in aggressive youth, we expected a negative relationship between levels of the different facets of IA and thickness of the aforementioned areas.

## Methods

This study was approved by The Baylor College of Medicine Institutional Review Board. Study participants were recruited from the child and adolescent outpatient psychiatric clinic at Texas Children's Hospital in Houston between January 2016 and October 2019, whereas HCs were recruited through Craigslist. Parents/legal guardians of youth who met study criteria as determined by a phone screen were approached for participation. Research staff explained the study and study procedures to the parental/legal guardians and children/adolescents. Written informed consent from a parent/legal guardian and written assent from the youth were obtained before any study procedure was initiated.

### Study Participants

Study participants included females and males, 7–17 years with a diagnosis of PBD and no history of psychiatric and neurologic disorder. Suitability for MRI scanning was also required for all the youths to be included. Exclusion criteria for youth with PBD were: (i) presence of an eating disorder, ADHD and anxiety disorders without comorbid PBD; (ii) comorbid substance use disorder; (iii) intellectual disability; (iv) comorbid autism spectrum disorder; (v) severe neurological conditions. Youth were diagnosed with BD type I and BD, type II per DSM-5 criteria; a diagnosis of BD, not otherwise specified (BDNOS) was made per the Course and Outcome of Bipolar Youth (COBY) research criteria (40).

### Clinical Assessments

Study participants were assessed using the 7.0.1 version of the Mini International Neuropsychiatric Interview-Kid-screen (MINI-KID) and the parent MINI-KID (41), and Wechsler Abbreviated Scale of Intelligence – II (WASI-II) (42). The MINI-KID and parent MINI-KID are structured interviews widely used to determine psychiatric diagnoses and have been updated to reflect DSM-5 diagnostic criteria. The WASI– II (WASI-II) (42) was used to determine age- and sex-corrected general intelligence (composite IQ score).

### Psychopathological Assessment

Severity of psychopathology was evaluated with the clinician rated 17-item Children's Depression Rating Scale (CDRS) for depressive symptoms (43) and clinician rated Young Mania Rating Scale (YMRS) for manic symptoms (44). YMRS scores were interpreted as follows: absence (0–12), minimal (13–20), mild (20–26), moderate (26–38),and severe (>38) manic symptoms, and CDRS scores are as follows: absence/minimal depressive symptoms (0 to 28), borderline (29–39) or frank depression (≥40) (43, 44). IA was assessed through the Aggression Questionnaire (AQ) (45), a 29-item scale composed by four subscales that measure four aspects of aggressive behaviors: hostility (AQ-hostility), anger (AQ-anger), verbal aggression (AQ-verbal aggression), physical aggression (AQ-physical aggression). The AQ provides also a total score (AQ-total).

### Cortical Thickness

All study participants underwent the same imaging protocol, which included a whole-brain T1-weighted scan acquired using a 3.0 T Siemens Trio scanner. Whole-brain T1-weighted images were obtained in the sagittal plane using the following sequence: TE/TR = 3.68/8.1 ms, matrix 256 × 256 × 180, voxel-size 1 × 1 × 1 mm^3^. Acquisition time lasted about 5 min. Cortical thickness was computed for 34 bilateral Desikan-Killiany (DK) atlas regions (46), using FreeSurfer 6.0 standard, automated cortical reconstruction pipeline (http://surfer.nmr.mgh.harvard.edu/). The processing steps were as follows: (i) Removal of non-brain tissue and transformation of the T1-weighted scans into the Talairach space (ii). Segmentation of subcortical white matter and gray matter anatomical volumes (47). (iii) Motion correction and non-uniform intensity normalization (48). (iv) Gray/white matter tessellation, topology correction (49) and intensity gradient-based surface deformation to generate gray/white and gray/cerebrospinal fluid surface models (49–51). The resulting surface models were then inflated and registered to a spherical surface atlas, allowing parcellation of cortical regions of interest (50, 52–54). Finally, regional cortical thicknesses were computed by taking the mean of the white-pial distance at all vertices within each parcellated region (26).

### Statistical Analyses

#### Demographics and Clinical Characteristics

Shapiro Wilk's test was used to check for continuous dependent variables' distributions for each group separately. In case of normal distribution, multiple *t*-tests for continuous variables (i.e., age, IQ), were performed to assess differences in the sociodemographic and clinical characteristics between groups. In case of non-normal distribution, Mann-Whitney tests were performed. In regards to nominal variables (i.e., gender, race/ethnicity), chi-square tests were performed.

#### Differences in Psychopathology and Cortical Thickness

Shapiro Wilk's test was used to check for continuous dependent variables' distributions for each group separately. In case of normal distribution, *t*-tests were performed to investigate differences in psychopathology and cortical thickness between groups. Otherwise, Mannn-Whitney tests were performed. In each test, the two groups (i.e., PBD, HC) were used as independent variables and rating scales assessing severity of psychopathology, i.e., YMRS total score, CDRS total score, AQ-hostility, AQ-anger, AQ-verbal aggression, AQ-physical aggression, AQ-total scores, and cortical thickness of discrete cerebral areas were used as dependent variables. Age, gender and IQ were used as covariates of no interest. Bonferroni correction was applied for multiple comparisons. Specifically, regarding cortical thickness, significance of the *p*-value was set at *p* = 0.0007 (*p* = 0.05/68). For the rating scale scores, the significance of the *p*-value was set at *p* = 0.007(*p* = 0.05/7).

#### Relationship Between Psychopathology and Cortical Thickness

In order to investigate the bi-directional relationships between neurobiological, and psychopathological measures in youth with PBD, canonical correlation analyses (CCAs) were performed. CCAs represent an approach which identifies relationships between two canonical (latent) variates, one representing a set of independent variables (also called predictor variables), the other a set of dependent variables (also called criterion variables). The CCA is optimized such that the linear correlation between the two latent variates is maximized. In other words, CCA finds the eigenvalues and the corresponding eigenvectors of a certain function of covariances. The maximum eigenvalue of the function of covariances is the maximum canonical correlation of the first independent variate and the first dependent variate, and the second largest eigenvalue is the second largest canonical correlation, and so on. Separate sets of coefficients or weights are applied to the predictor and criterion variables to form the linear combinations.

**Figure 1:**
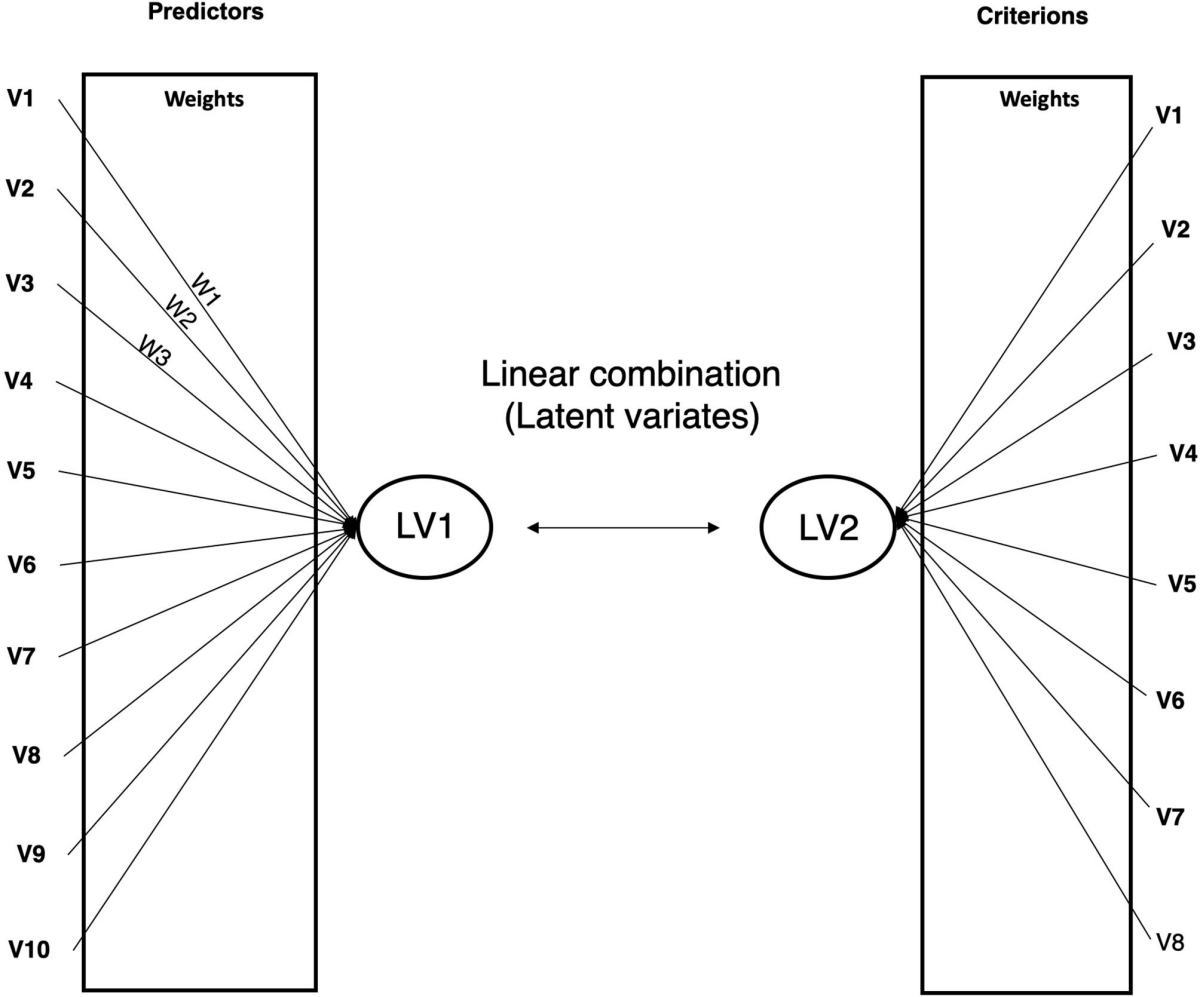
Graphical representation of canonical correlation analysis. LV, latent variate. W, variable's weight.

These weights and related statistics, known as loadings, are used to interpret the results of canonical analysis. Therefore, interpretation of what the latent variates represent and how they are related to each other can be determined by the weighted loadings of individual measures on the latent structure, much like principal components analysis. A graphical representation of CCA is provided in Figure 1.

In this work, variables differentiating youth with PBD from HC entered the CCA, and then, a CCA was conducted for psychopathological measurement. Signs of loadings were used in order to interpret how scores on individual measures related to the latent variates. Therefore, loadings indicate what aspect of psychopathology is captured in each analysis, the neurobiological characteristics with which they are associated, and the nature of the relationship between them. In this view a positive value of a loading indicates higher scores on the individual measures whereas a negative value indicates lower scores on individual measures. Only moderate-strong loadings (beyond −0.2 or 0.2) were taken into account.

#### Effect of Additional Clinical Variables

As comorbid conditions, medications and duration of illness might affect either IA and brain structure (55–57), additional analyses were conducted to assess the impact of such variables. In order to investigate the effects of comorbidity, the aforementioned *t*-tests were re-run after subtracting study participants with attention deficit hyperactivity disorder (ADHD; *N* = 7), obsessive-compulsive disorder (OCD; *N* = 1), panic disorder (*N* = 1), anxiety disorder (*N* = 4). To investigate the effect of medications, the PBD group was split in those with or without antidepressants (PBDAD, *N* = 12; PBDNAD, *N* = 11 respectively), antipsychotics (PBDAP, *N* = 10; PBDNAP, *N* = 13 respectively), mood stabilizers (PBDMS, *N* = 11; PBDNMS, *N* = 12 respectively) and mixed monoamine reuptake inhibitors (PBDMARI, *N* = 8; PBDNMARI, *N* = 15 respectively). Then multiple one-way analyses of variance (ANOVAs) were run. In each ANOVA, the aforementioned groups (PBDAD and PBDNAD; PBDAP and BBPNAP; PBDMS and PBDNMS; PBDMARI and PBDNMARI) were used as independent variables and rating scales assessing severity of psychopathology, i.e., YMRS total score, CDRS total score, AQ-hostility, AQ-anger, AQ-verbal aggression, AQ-physical aggression, AQ-total scores, and cortical thickness of discrete cerebral areas were used as dependent variables. Age, gender and IQ were used as covariates of no interest. Bonferroni correction was applied for multiple comparisons. As regards the effect of years of disease, such variable was included in the CCA in the first set of variates.

## Results

Sixty-two study participants entered the study. Eight subjects were excluded because they did not fulfill inclusion criteria. Additionally, other 6 subjects were excluded because of motion artifacts. Therefore, the final sample consisted of 46 study participants.

Shapiro-Wilks test showed that variables analyzed have a normal distribution, with the exception of IQ in PBD (W = 0.900; df = 23; *p* = 0.03). Therefore, Mann–Witney test was performed for investigating between-group differences in IQ. For the other continuous variables, *t*-tests were performed.

### Demographics and Clinical Characteristics

Study participants did not show differences in age, gender, race/ethnicity and IQ. More than a half of the study participants had at least one comorbidity. The most frequent comorbid condition was ADHD, followed by anxiety disorders. All study participants were on medications with most participants being on antidepressants and mood stabilizers (see Table 1).

**Table 1:** Sociodemographic and clinical characteristics of youth with PBD and HC.

	**PBD (*N* = 23)**	**HC (*N* = 23)**	***F* or χ^2^**	***p-value***
**Demographics**				
Age (y), mean ± SD	12.26 ± 3.24	12.00 ± 3.25	0.07	0.79
Female, n (%)	15 (65.20)	13 (56.50)	0.37	0.55
Race, n (%)				
*Asian*	0 (0.00)	3 (13)		
*African-American*	2 (8.7)	4 (17.4)	4.34	0.11
*Caucasian*	21 (91.3)	16 (69.6)		
Ethnicity				
*Hispanic*	5 (21.7)	1 (4.3)	3.07	0.08
I.Q., mean ± SD	103.22 ± 13.36	98.61 ± 15.70	188^*^	0.10
**Clinical**				
Year ill (y), mean ± SD	3.30 ± 2.30	–	–	–
PBD type				
*Type 1*	15 (65.20)	–	–	–
*Type II*	1 (4.30)			
*Not otherwise specified*	7 (30.40)	–	–	–
Comorbidity, n (%)				
*None*	10 (43.50)	–	–	–
*ADHD*	7 (30.40)	–	–	–
*OCD*	1 (4.30)	–	–	–
*Panic Disorder*	1 (4.30)	–	–	–
*Anxiety Disorder*	4 (17.40)	–	–	–
Current pharmacotherapy, n (%)				
*AD*	12 (52.20)	–	–	–
*AP*	10 (43.50)	–	–	–
*MS*	11 (47.80)	–	–	–
*BDZ*	0 (0.00)	–	–	–
*MARI*	8 (34.80)	–	–	–

*Significant p-values (p < 0.05) are indicated in bold, trend-level p-values (p < 0.07) are indicated in italics. PBD, youth with bipolar disorder; HC, healthy controls; ADHD, attention deficit hyperactivity disorder; OCD, obsessive-compulsive disorder. AD, antidepressant; AP, antipsychotic; MS, mood stabilizer; BDZ, Benzodiazepine; MARI, mixed monoamine reuptake inhibitor*.

* *This value refers to the Mann Wihtney U*.

### Psychopathology

Youth with PBD showed mild levels of both manic and depressive symptoms. Presence of both manic and depressive symptoms, even though with mild intensity, are indicative of the presence of a mixed state in the PBD group. As regards IA, youth with PBD showed higher scores on the AQ-anger and AQ-hostility subscales and higher scores on the AQ-total than HC (see Table 2).

**Table 2:** Clinical scales in youth with PBD and HC.

	**PBD (*N* = 23)**	**HC (*N* = 23)**	***F***	***p-value***
**Test**				
CDRS	33.96 ± 13.75	17.39 ± 4.11	7.76	**<0.001**
YMRS	13.83 10.16	2,70 ± 3.43	5.09	**0.001**
AQ				
*Hostility, mean ±* SD	20.30 ± 7.60	11.13 ± 5.55	6.23	**0.001**
*Anger, mean ±* SD	19.35 ± 6.30	12.39 ± 3.59	7.27	**<0.001**
Verbal aggression, *mean ± SD*	13.61 ± 4.71	10.22 ± 5.43	3.47	0.016
Physical aggression*, mean ±* SD	20.00 ± 7.94	14.52 ± 5.11	2.54	0.055
*Total, mean ±* SD	29.09 ± 14.41	11.74 ± 11.18	6.55	**<0.001**

*Significant p-values (p < 0.05) are indicated in bold, trend-level p-values (p < 0.07) are indicated in italics. PBD, youth with bipolar disorder; HC, healthy controls. CDRS-R, Children Depression Rating Scale-Revised; YMRS, Young Mania Rating Scale; AQ, Aggression Questionnaire*.

### Cortical Thickness

**Figure 2:**
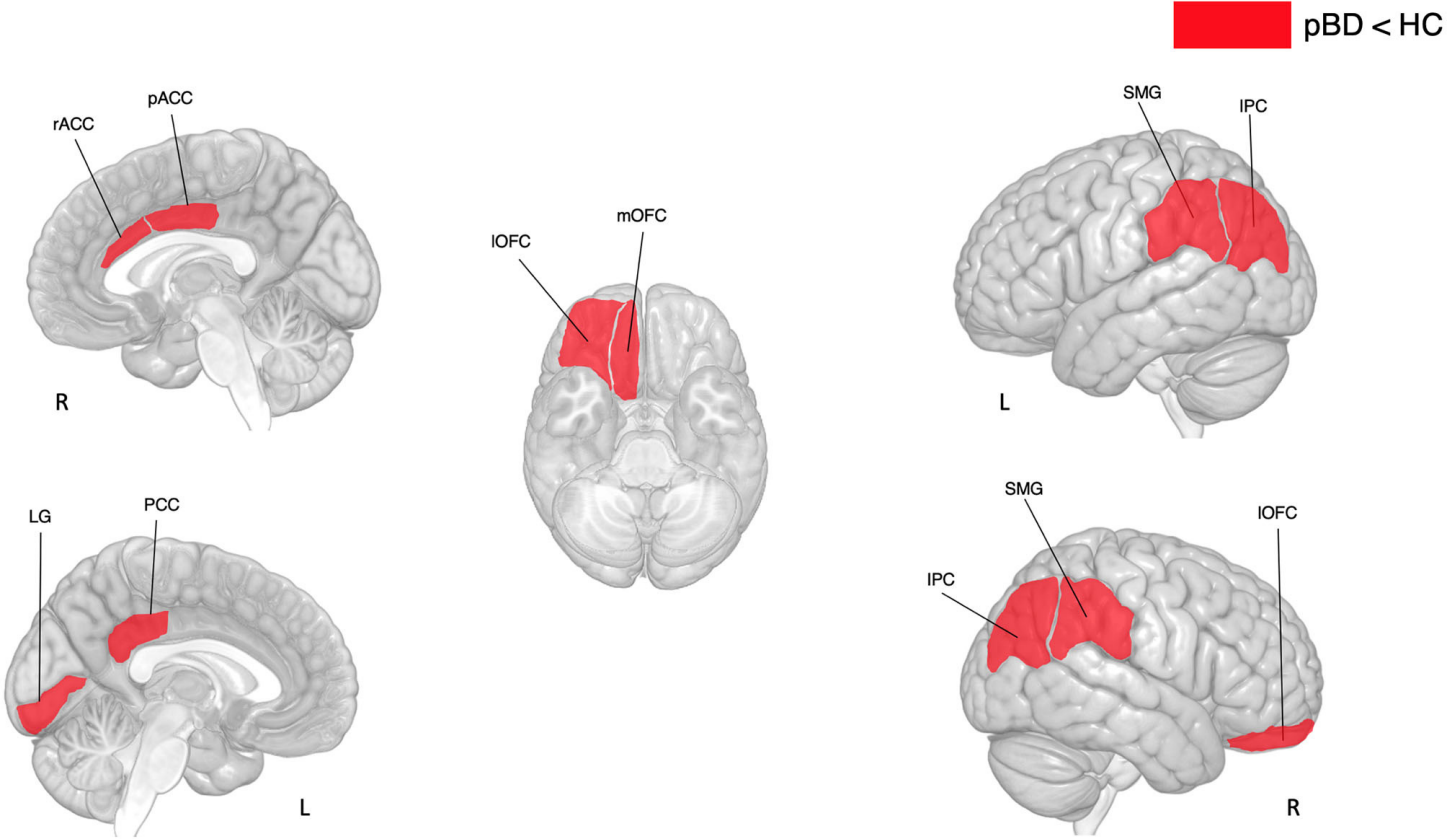
Differences in cortical thickness between subjects with pBD and HC. pBD, pediatric bipolar disorder; HC, healthy controls. IPC, inferior parietal cortex; SMG, supramarginal gyrus; LG, lingual gyrus; lOFC, lateral orbitofrontal cortex; mOFC, medial orbitofrontal cortex; pACC, posterior anterior cingulate cortex; PCC, posterior cingulate cortex; rACC, rostral anterior cingulate cortex.

*T*-tests revealed that in comparison to the HC, youth with PBD have reduced thickness in frontal (right rostral anterior cingulate, right caudal anterior cingulate, right lateral orbitofrontal, right medial orbitofrontal), parietal (left and right inferior parietal, left posterior cingulate, left and right supramarginal) and occipital (left lingual) areas (see Table 3 and Figure 2).

**Table 3:** Cortical thickness in youth with PBD and HC.

	**PBD (*N* = 23)**	**HC (*N* = 23)**	***F***	***p-value***
Cortical areas				
**Frontal**				
*R Rostral anterior cingulate*	3.10 ± 0.27	3.16 ± 0.35	8.69	**<0.0001**
*L Rostral anterior cingulate*	2.99 ± 0.29	3.09 ± 0.24	2.59	0.051
*R Caudal anterior cingulate*	2.62 ± 0.22	2.78 ± 0.24	12.63	**<0.0001**
*L Caudal anterior cingulate*	2.78 ± 0.33	2.77 ± 0.11	3.11	0.025
*R Isthmus cingulate*	2.55 ± 0.16	2.64 ± 0.18	2.69	0.044
*L Isthmus cingulate*	2.53 ± 16	2.61 ± 0.21	2.76	0.040
*R Caudal middle frontal*	2.61 ± 0.14	2.66 ± 0.12	2.22	0.126
*L Caudal middle frontal*	2.62 ± 0.13	2.64 ± 0.09	1.70	0.168
*R Lateral orbitofrontal*	2.78 ± 0.20	2.89 ± 0.17	8.77	**<0001**
*L Lateral orbitofrontal*	2.85 ± 0.22	2.25 ± 0.12	4.02	0.008
*R Medial orbitofrontal*	2.61 ± 0.20	2.70 ± 0.23	7.29	**0.0002**
*L Medial orbitofrontal*	2.59 ± 0.26	2.60 ± 0.19	3.44	0.016
*R Pars opercularis*	2.76 ± 0.18	2.64 ± 0.17	2.82	0.037
*L Pars opercularis*	2.80 ± 0.17	2.83 ± 0.12	0.883	0.132
*R Pars orbitalis*	2.86 ± 0.24	2.96 ± 0.20	2.24	0.082
*L Pars orbitalis*	2.91 ± 0.25	2.98 ± 19	1.54	0.208
*R Pars triangularis*	2.65 ± 0.21	2.63 ± 0.18	1.87	0.134
*L Pars triangularis*	2.64 ± 0.17	2.69 ± 0.14	1.86	0.136
*R Precentral*	2.54 ± 0.18	2.66 ± 0.10	2.15	0.092
*L Precentral*	5.13 ± 0.30	5.36 ± 0.17	2.47	0.059
*R Rostral middl efrontal*	2.49 ± 0.18	2.48 ± 0.12	3.97	0.008
*L Rostral middle frontal*	2.47 ± 0.17	2.46 ± 0.11	5.07	0.002
*R Superior frontal*	2.85 ± 0.20	2.93 ± 0.08	3.90	0.009
*L Superior frontal*	2.83 ± 0. 18	2.88 ± 0.13	3.84	0.010
*R Frontal pole*	2.89 ± 0.32	2.90 ± 0.33	1.81	0.053
*L Frontal pole*	2.89 ± 0.32	2.81 ± 0.34	2.16	0.090
**Temporal**				
*R Bank STS*	2.75 ± 0.24	2.87 ± 0.20	2.68	0.045
*L Bank STS*	2.77 ± 0.19	2.77 ± 0.11	2.11	0.097
*R Entorhinal*	3.45 ± 0.47	3.43 ± 0.41	0.73	0.577
*L Entorhinal*	3.36 ± 0.37	3.48 ± 0.33	1.94	0.122
*R Fusiform*	2.86 ± 0.20	2.92 ± 0.13	2.38	0.067
L Fusiform	2.91 ± 0.17	3.00 ± 0.13	3.97	0.008
*R Superior temporal*	2.99 ± 0.25	3.10 ± 0.20	2.79	0.039
*L Superior temporal*	2.93 ± 0.25	3.04 ± 0.12	2.01	0.111
*R Middle temporal*	3.08 ± 0.24	3.15 ± 0.12	1.73	0.161
*L Middle temporal*	2.99 ± 0.24	3.11 ± 0.16	2.14	0.094
*R Inferior temporal*	2.88 ± 0.20	2.88 ± 12	1.83	0.141
*L Inferior temporal*	2.88 ± 0.22	2.96 ± 0.14	1.68	0.199
*R parahippocampal*	2.90 ± 0.25	3.00 ± 0.21	2.19	0.087
*L parahippocampal*	2.99 ± 0.32	3.08 ± 0.29	1.88	0.132
*R Temporal pole*	3.71 ± 0.31	3.77 ± 0.33	0.69	0.604
*L Temporal pole*	3.64 ± 0.35	3.80 ± 0.28	0.82	0.518
*R Transversal temporal*	2.70 ± 0.30	2.84 ± 0.24	2.39	0.066
*L Transversal temporal*	2.70 ± 0.26	2.83 ± 0.23	5.87	0.001
*R Insula*	3.17 ± 0.21	3.22 ± 0.19	3.21	0.022
*L Insula*	3.21 ± 0.16	3.30 ± 0.21	2.70	0.044
**Parietal**				
*R Superior parietal*	2.22 ± 14	2.29 ± 0.13	4.64	0.004
*L Superior parietal*	2.26 ± 0.16	2.31 ± 13	4.07	0.007
*R Inferior parietal*	2.58 ± 0.13	2.62 ± 0.15	12.05	**<0.0001**
*L Inferior parietal*	2.56 ± 0.20	2.58 ± 0.16	6.27	**0.0005**
*R Paracentral*	2.50 ± 0.22	2.64 ± 0.17	1.98	0.116
*L Paracentral*	2.52 ± 0.20	2.63 ± 0.18	3.18	0.025
*R Posterior cingulate*	2.64 ± 0.19	2.71 ± 1.00	2.87	0.035
*L Posterior cingulate*	2.65 ± 0.21	2.74 ± 0.17	6.49	**0.0004**
*R Postcentral*	*2.18 ± 0.17*	*2.21 ± 0.18*	*2.83*	*0.010*
*L Postcentral*	5.13 ± 0.30	5.36 ± 0.17	2.35	0.070
*R Precuneus*	2.56 ± 0.16	2.61 ± 0.12	5.16	0.002
L Precuneus	2.56 ± 16	2.60 ± 0.14	11.33	0.001
*R Supramarginal*	2.70 ± 0.14	2.77 ± 0.14	11.01	**<0.0001**
*L Supramarginal*	2.71 ± 0.15	2.82 ± 0.15	8.82	**<0.0001**
**Occipital**				
R Cuneus	2.01 ± 0.14	2.10 ± 0.17	4.09	0.007
*L Cuneus*	2.03 ± 19	2.03 ± 0.15	5.37	0.001
*R lateral occipital*	2.27 ± 0.13	2.32 ± 0.12	1.92	0.126
*L lateral occipital*	2.22 ± 0.15	2.25 ± 12	2.93	0.032
*R lingual*	2.21 ± 0.15	2.24 ± 0.16	5.38	0.001
*L lingual*	2.18 ± 0.17	2.21 ± 0.14	8.15	**<0.0001**
*R pericalcarine*	1.75 ± 0.15	1.79 ± 0.09	1.71	0.166
*L pericalcarine*	1.80 ± 0.20	1.78 ± 0.13	1.25	0.305

*Significant p-values (p < 0.05) are indicated in bold, trend-level p-values (p < 0.07) are indicated in italics. PBD, youth with bipolar disorder; HC, healthy controls*.

### Relationship Between Cortical Thickness and Psychopathology

**Figure 3:**
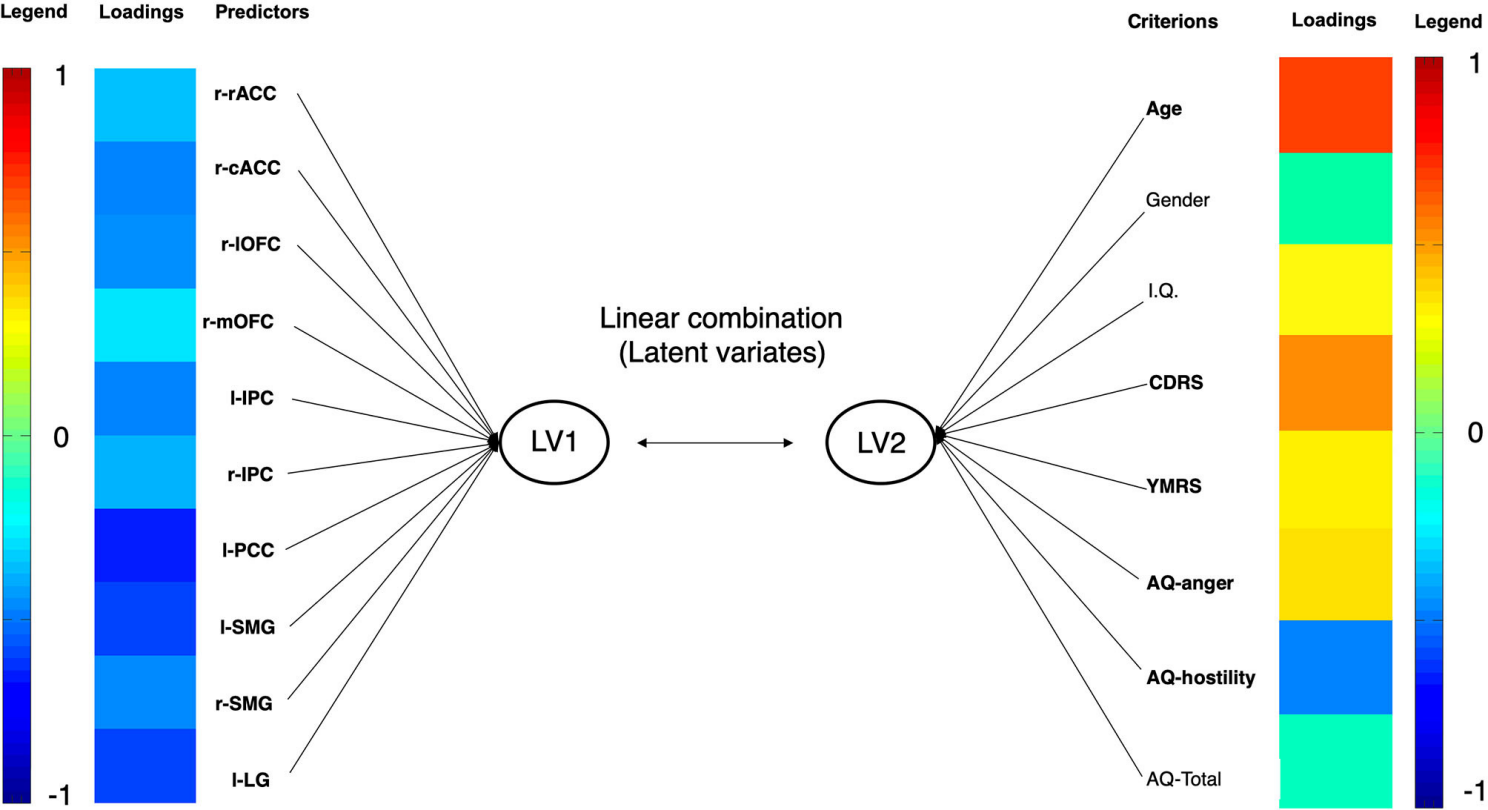
Graphical representation of CCA in youth with PBD. Each variable's loading strength is represented with a color. As the loading approaches the value of −1, the loading color will approach the color deep blue. As the loading approaches the value of 1, the loading color will approach the color red. PBD, pediatric bipolar disorder. IPC, inferior parietal cortex; SMG, supramarginal gyrus; LG, lingual gyrus; lOFC, lateral orbitofrontal cortex; mOFC, medial orbitofrontal cortex; pACC, posterior anterior cingulate cortex; PCC, posterior cingulate cortex; rACC, rostral anterior cingulate cortex. LV, latent variate.

Differences between youth with PBD and HC involve 5 psychopathological variables (i.e., YMRS, CDRS, AQ-anger; AQ-hostility. AQ-total) and the cortical thickness of 10 brain areas (i.e., right rostral anterior cingulate, right caudal anterior cingulate, right lateral orbitofrontal, right medial orbitofrontal, left and right inferior parietal, left posterior cingulate, left and right supramarginal, left lingual). Therefore, cortical thicknesses of the aforementioned brain areas were included in the first set, whereas psychopathological measures were included in the second set. Age, sex, and IQ entered the model in the second set in order to control the results for the effect of these variables. The CCA revealed that only the correlation between the first pair of variates was significant (see Table 4). In the CCA, thicknesses of the right rostral anterior cingulate, right caudal anterior cingulate, right lateral orbitofrontal, right medial orbitofrontal, left and right inferior parietal, left posterior cingulate, left and right supramarginal, left lingual negatively correlated with the latent variate. In regards to psychopathological measures, both CDRS and YMRS scores positively correlated with the latent variable, with the latter showing greater association than the former. In regards to IA, AQ-anger positively correlated with the latent variate, whereas AQ-hostility negatively correlated with it. Within the demographic characteristics, age showed a strong positive correlation with the latent variate. The other variables showed a weak relationship with the latent variate (see Figure 3).

**Table 4:** Significance of CCA variate pairs in youth with PBD.

**CCA**	**Canonical correlation**	**Squared canonical correlation**	**Eigenvalue**	**Wilk's Lambda**	***F***	***p-*value**
Pair1	0.99	0.98	86.12	<0.001	4.34	**0.004**

*Significant p-values (p < 0.05) are indicated in bold, trend-level p-values (p < 0.07) are indicated in italics. PBD, youth with bipolar disorder*.

### Effect of Other Clinical Variables

After removing youth with ADHD, OCD, panic disorder and anxiety disorder, *t*-tests regarding psychopathology and cortical thickness did not change. In regard to medications, one-way ANOVAs did not show any differences amongst groups with regard to psychopathology and cortical thickness. Regarding the impact of the duration of illness on the results found, the CAA revealed that only the correlation between the first pair of latent variates was significant (canonical correlation, 0.99; squared canonical correlation, 0.99; eigenvalue, 95.07; Wilk's lambda <0.001; *p* = 0.002). Duration of illness' loading on the latent variate reached a value of 0.158, meaning a weak correlation with the latent variate.

## Discussion

Results might be summarized as follows: (i) youth with PBD showed heightened levels of anger and hostility compared with HC. (ii) youth with PBD showed cortical thinning in prefrontal and parietal cortices. (iii) in youth with PBD, thinning of these cortices was associated with higher levels of anger and lower levels of hostility.

These findings are in line with those reporting heightened anger and hostility (58, 59) and low level of interpersonal violence despite the high levels of incarceration in youth with PBD (60, 61). Additionally, these findings replicate those reporting altered thickness in ACC and PCC (62), OFC (63, 64), inferior parietal cortex (IPC) (65), lingual cortex (LC) and supramarginal gyrus (SMG) (66) in youth with PBD even though cortical thinning has been found in other regions, such as the precentral and superior frontal (67) cortices, or temporal regions (62). Indeed, discrepancies with these findings might be due to the small sample size of our study, or the sample selection of the other studies, which mainly included young adults. Further studies, with larger sample sizes and narrower inclusion criteria are necessary to define alterations in cortical thickness in youth with PBD.

Thinner cortical areas in youth with PBD, i.e., ACC, PCC, OFC, IPC, SMG, LC, are part of two interconnected networks involved in emotion regulation and impulse control. The rostral part of the ACC and the medial and lateral parts of the OFC belong to the affective network (AN) (68–70). The OFC and ACC are densely interconnected with the amygdala and the other subcortical limbic and paralimbic regions through specific feedback and feedforward pathways that serve to convey attention to motivationally relevant stimuli and provide information about the emotional salience or significance of external stimuli in order to rapidly perceive reward contingency (35). Furthermore, both areas participate in the voluntary regulation of emotionally relevant states (36). The other regions belong to a broad network involved in the initiation, maintenance and control of goal directed behavior, and indirectly implicated in impulse control (37, 38). Such “control network” embeds several functions including attention, working memory, response selection, response inhibition, and task switching, that are activated over time in order to prevail against distraction and respond quickly to unpredictable demands that arise during performances (39). This network can be subdivided in two functionally distinct, and partially overlapping networks: the frontoparietal network (FPN) and the cingulo-opercular network (CON). The CON includes the dorsal1 ACC, the PCC, and the LG and contributes to the flexible control of human goal-directed behavior through the stable, across-trial implementation of task sets in downstream sensorimotor processors (37). On the other hand, the FPN includes the IPC, middle cingulate cortex, and supports control initiation and provides flexibility by adjusting control in response to feedback (38).

The CCA demonstrated that the indirect evidence of dysfunction in such networks, i.e., cortical thinning, is correlated with both CDRS and YMRS mean scores. Accordingly, alteration of the AN has been related to manic, depressive, and mixed states, in which depressive and manic phases co-occur (71, 72). Furthermore, the core clinical features of mixed states i.e., irritability, impulsivity and anger, have been related to alterations of both FPN and CON (73, 74). The CCA also show that cortical thinning of areas belonging to the AN, FPN and CON are associated with higher levels of anger, whereas, an opposite pattern was found with hostility. Anger and hostility are considered as two distinct constructs. Anger is seen as an emotional state consisting of physiological, phenomenological and expressive behavioral variables (6, 7) whereas hostility is regarded as more of a cognitive construct (75). Hostility involves cognitive variables of cynism, mistrust and denigration (76), which are mainly driven by a tendency toward viewing others' actions as hostile and purposeful when their intention is unclear, i.e., the hostile attributional bias (8). Activation of the AN, and more specifically, selective hyperactivation of the OFC, has been related to the development of preference for evaluating ambiguous social cues as hostile (77). On the other hand, activation of FPN and OCN has been related to detection of threat (78) and attention bias toward threat (79). Therefore, greater thickness of areas related to AN, FPN, and CON, possibly reflecting greater activity of these networks, might produce greater bias toward negative stimuli and greater alert toward the others, i.e., greater hostile attributional bias. This might turn in greater levels of hostility.

On the other hand, negative correlations between anger and thickness of cortical areas belonging to AN, FPN, and CON might reflect poor activity of these networks as anger outbursts increase. As mentioned before, altered activity of cortical areas belonging to AN are involved in emotion regulation. Poor emotion regulation does not allow for the inhibition of prepotent emotional impulses coming from subcortical areas, such as the amygdala (80). Poor activity in the FPN and CON might facilitate the transition of prepotent emotionally states into action through failure of response inhibition processes., i.e., the capability of withholding or canceling routinized, ongoing, or prepotent responses to enable goal-oriented behaviors (81, 82). Altered emotion regulation and response inhibition might allow emotional negativity to bypass deliberate processing and lead to anger outbursts.

This work has several limitations. First, the small sample size limits the generalizability of the results found. Therefore, larger sample sizes are needed to clearly investigate the relationship between cortical thickness and impulsive aggression in youth with PBD. Second, a narrower age range in youth is needed to investigate the interplay between brain regional developmental trajectories and psychopathology. The broad age range in the present study did not account for brain regional developmental maturation differences. In fact, regional gray matter thickness' plateau varies with age, spanning from 7 to 11 years old, and follows a maturational pattern that could be linear, quadratic or cubic (83). Additional studies with sample sizes including only children or adolescents are needed to investigate whether the present findings are applicable to either children and adolescents. Third, the effect of possible confounding variables might have interfered with the results found. Such variables have been proved to modify brain and behavior and include, but are not limited to specific medications (84–86), childhood trauma (87, 88) or predominant polarity (89). Specific work on the effect of these variables on the neural bases of impulsive aggression are warranted to clarify their impact.

## Conclusions

This preliminary work highlights that altered thickness in areas belonging to the AN, PFN and CON are related to the different facets of impulsive aggression. Specifically, results suggest that hyperactivation of the AN, FPN, and CON might result in greater levels of hostile attributional bias, thus increasing levels of hostility. On the other hand, hypoactivation of the aforementioned structures are related to affective dysregulation and behavioral disinhibition, which can result in anger outbursts. Future research and larger samples, using different MRI techniques, are needed to clarify dysfunctional patterns underlying impulsive aggression and its multiple facets in youth with PBD. Novel treatments specifically restoring balance in the activity of such networks are warranted to prevent the development of hostile behaviors and anger outbursts in children and adolescents with PBD.

## Data Availability Statement

The raw data supporting the conclusions of this article will be made available by the authors upon request, without undue reservation.

## Ethics Statement

The studies involving human participants were reviewed and approved by Baylor College of Medicine Institutional Review Board. Written informed consent to participate in this study was provided by the participants' legal guardian/next of kin.

## Author Contributions

KS, GS, and AS designed the study. AS, SK, and JS acquired data. CV and MDN analyzed the data, together with KS and SK. AS, AR, JCS, and GS drafted the manuscript. KS, AR, CV, MDN, and JCS revised the manuscript. All the authors approved the final version of the manuscript.

## Conflict of Interest

The authors declare that the research was conducted in the absence of any commercial or financial relationships that could be construed as a potential conflict of interest.
